# Case Report: Corpus Callosotomy in a Cat With Drug-Resistant Epilepsy of Unknown Cause

**DOI:** 10.3389/fvets.2021.745063

**Published:** 2021-09-29

**Authors:** Daisuke Hasegawa, Rikako Asada, Satoshi Mizuno, Yoshihiko Yu, Yuji Hamamoto, Shinichi Kanazono

**Affiliations:** ^1^Laboratory of Veterinary Radiology, Faculty of Veterinary Science, Nippon Veterinary and Life Science University, Tokyo, Japan; ^2^The Research Center for Animal Life Science, Nippon Veterinary and Life Science University, Tokyo, Japan; ^3^Veterinary Medical Teaching Hospital, Nippon Veterinary and Life Science University, Tokyo, Japan; ^4^Neurology and Neurosurgery Service, Veterinary Specialists & Emergency Center, Saitama, Japan

**Keywords:** feline, corpus callosotomy, drug-resistant epilepsy, epilepsy surgery, quality of life

## Abstract

A 2-month-old, intact male domestic shorthair cat with dullness, bilateral central blindness, and recurrent epileptic seizures was presented to a local clinic. Seizures were the generalized myoclonic and tonic-clonic type. Phenobarbital was initiated and maintained; however, seizures were not controlled. Other anti-seizure drugs, including levetiracetam, zonisamide, and diazepam, also provided insufficient seizure control with seizures occurring hourly to daily. By 8 months of age, the cat displayed non-ambulatory tetraparesis and deep somnolence. Magnetic resonance imaging (MRI), cerebrospinal fluid analysis, and pre- and post-prandial total bile acid analyses were unremarkable. Scalp electroencephalography (EEG) revealed central dominant but generally synchronized spikes and multiple spikes. The cat was diagnosed with drug-resistant epilepsy of unknown cause and was included in a clinical trial of epilepsy surgery. Given the unremarkable MRI and bilateral synchronized EEG abnormalities, a corpus callosotomy was performed at 12 months of age, and partial desynchronization of spikes was confirmed on EEG. Incomplete transection was found in the genu of the corpus callosum on postoperative MRI. After surgery, the mental status and ambulation clearly improved, and seizure frequency and duration were remarkably reduced. Recheck with follow-up EEG and MRI were performed at 3, 6, and 12 months after surgery. Scores of activities of daily living and visual analog scales including cat's and owner's quality of life had also improved considerably. This case report is the first documentation of the one-year clinical outcome of corpus callosotomy in a clinical feline case with drug-resistant epilepsy.

## Introduction

The corpus callosum (CC) comprises the largest commissural fibers connecting both cerebral hemispheres, lying in the center of the forebrain longitudinally. Corpus callosotomy (CCT), i.e., dividing hemispheres (split-brain) by disconnecting CC, is a type of palliative epilepsy surgery mainly applied to human patients with infantile epileptic encephalopathy and drug-resistant generalized epilepsy with atonic, absence, myoclonic, and tonic-clonic seizures (GTCS). In particular, the efficacy of CCT has been established in atonic or astatic seizures, also known as drop attack, that shows sudden astasia causing head injury (1–3).

In veterinary medicine, the surgical procedure for CCT in normal dogs has been reported by Bagley et al. in 1995 (4), and the effect of CCT for epilepsy had been experimentally studied in feline seizure models such as kindling between the 1960s and 1990s (5–8). However, its clinical application and therapeutic outcome has not been reported so far. Here, we report the first client-owned feline case of drug-resistant epilepsy (DRE) of unknown cause undergoing CCT and the outcome after 1 year.

## Case Presentation

### History

A 2-month-old male domestic shorthair cat was presented to a local veterinary clinic with a complaint of recurrent generalized convulsions. The local veterinarian noticed the cat also had frequent facial twitching, ataxia, visual impairment, and slightly decreased mentation at the first consultation. Video clips recorded by the owner (Supplementary Video 1) showed generalized myoclonic seizures, which was momentary but clustered for 1 to a few minutes, and GTCS lasting about several tens of seconds to 1 minute. These seizures were initially observed several times per week; therefore, antiseizure drug (ASD) therapy with 1.0 mg/kg of phenobarbital (PB) every 12 h per os was initiated. The dose of PB was increased to 2.0 mg/kg q12h PO (achieved static serum level: 14.8 μg/ml) at 3 months of age due to insufficient seizure control. At this time, complete blood count, serum chemistry including fasted and postprandial ammonia and total bile acid, urinalysis, screening test of feline immunodeficiency virus and feline leukemia virus, cerebrospinal fluid (CSF) analysis including PCR for *Toxoplasma gondii* and feline corona virus, and low-field (0.4T) magnetic resonance imaging (MRI) of the brain were performed. All complementary tests were unremarkable. Due to severely increased seizure frequency to > 40 times daily (uncountable), the dose of PB was further increased (4.0 mg/kg q12 h PO), and zonisamide (8.0 mg/kg q12h PO), diazepam (DZP; 0.5 mg/kg q12 h PO) as well as levetiracetam (LEV; 20 mg/kg q8 h PO) were added. However, zonisamide was discontinued 3 days after the initiation due to the appearance of aphagia.

At 5 months of age, the cat was referred to a veterinary neurologist (SK) at the Veterinary Specialists Emergency Center (Saitama, Japan). Neurological examination revealed severe mental dullness, disorientation, difficulty in standing and walking, decreased postural reactions in all limbs, and bilateral menace response deficits with normal pupillary light reflexes. Appetite remained normal but the cat needed assisted feedings. Levetiracetam had been discontinued by the owner because the cat seemed to develop stupor after its administration. Doses of PB and DZP were maintained at 5.0 mg/kg q12 h PO (achieved static serum level: 27.1 μg/ml) and 0.8 mg/kg q12 h PO, respectively. Although the frequency and duration of GTCS decreased, myoclonic seizures and short tonic seizures still occurred 1–6 times per day (sz/d). Although other differential diagnoses at this point including inborn errors of metabolism, inherited neurodegenerative conditions, or immune-mediated limbic encephalitis could not be completely ruled out, further diagnostics such as the Tandem mass spectrometry and/or investigation of known antibodies such as anti-voltage-gated potassium channel and/or anti-leucine-rich glioma inactivated 1 receptor antibodies for limbic encephalitis were unavailable locally then. At 7 months of age, the cat was presumptively diagnosed as DRE of unknown cause with severely impaired quality of life (QOL), when the potential candidacy of the epilepsy surgery research project was discussed and the owner requested the candidacy to be evaluated.

The project team of veterinary epilepsy surgery (Supplementary Data 1.1) discussed this case based on the abovementioned history and data. All members considered this cat as a candidate for epilepsy surgery. Electroencephalography (EEG) and high-field MRI as presurgical evaluations were performed to determine the type of surgical procedure.

### Presurgical Evaluations and Decision of Surgical Procedure

**Figure 1:**
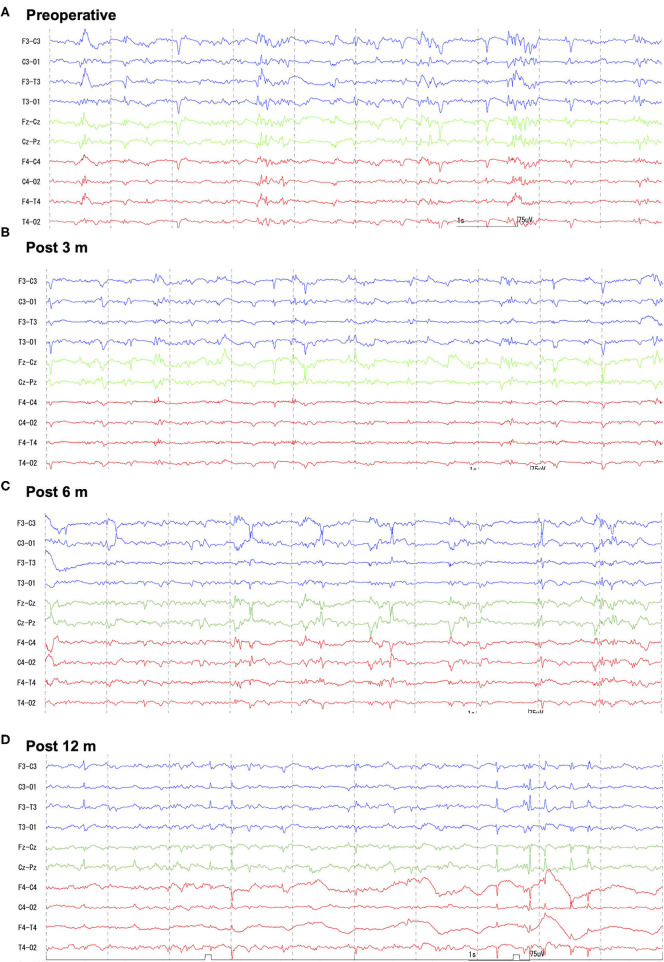
Preoperative and follow-up scalp EEGs. Preoperatively **(A)** generalized spikes and multiple spike complex were frequently observed with phase reversal at the central region (C3, Cz, C4). Follow-up EEGs [**(B)** 3-months; **(C)** 6-months; **(D)** 12-months after surgery] showed decreased spike frequency, and some small spikes were found to be limited to one hemisphere. However, large spikes were always bilaterally synchronized. Bipolar montages: blue traces are left (F3-C3, C3-O1, F3-T3, T3-O1), greens are midline (Fz-Cz, Cz-Pz), and reds are right (F4-C4, C4-O2, F4-T4, T4-O2). Sensitivity = 15 μV, Time constant = 0.1, High-cut filter = 60 Hz, AC filter = ON.

**Figure 2:**
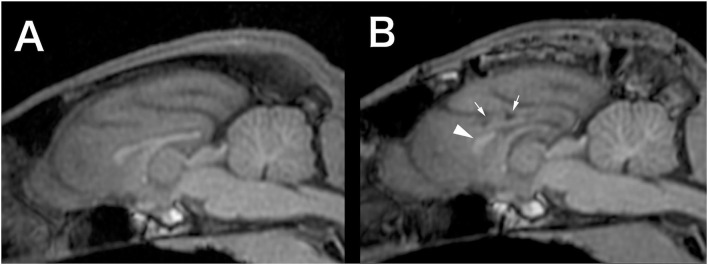
Preoperative **(A)** and 12-months postoperative **(B)** midline sagittal T1-weighted MRI (generated by multiplanar reconstruction from 3D-T1 data). In the postoperative image, the genu of the corpus callosum (arrowhead) left intact and necrotic lesions in the cingulate gyrus (small arrows), which was damaged during the surgery, were observed.

At 8 months of age, the cat was further referred to the Veterinary Medical Teaching Hospital of Nippon Veterinary and Life Science University (Tokyo, Japan) to undergo EEG and high-field MRI. At this point, seizure frequency increased reaching up to 7–10 sz/d, including GTCS. The cat was non-ambulatory and nearly stuporous but remained responsive to forced feeding, loud sounds, and nociceptive stimulation. Postural reaction deficits and bilateral menace deficits were noticed on neurological examination; no other abnormalities were recorded. Feeding, drinking, and urination had been fully assisted by the owner. Complete blood count and serum chemistry profile were re-evaluated and revealed mild dehydration, which was easily improved later by increasing the water intake. Scalp EEG under sedation (Supplementary Data 1.2) revealed frequent spikes and multiple spike complexes were observed on the central regions (C3, C4, Cz) dominantly but synchronized generally (Figure 1A). High-field MRI (3.0T) under general anesthesia (Supplementary Data 1.3) showed no appreciable structural abnormalities in the whole brain (Figure 2A). Cerebrospinal fluid analysis including cell count (0/μl; ref 0–5/μl), cytology (no cells observed), protein concentration (12.4 mg/dl; ref 0–25 mg/dl), glucose (126 mg/dl; ref 35–140 mg/dl), and electrolytes (Na^+^ 158; K^+^ 2.9; Cl^−^ 135 mEq/l) was also unremarkable. The serum level of PB at this point was 33 μg/ml. With these repeated MRI and CSF analysis, inherited neurodegenerative conditions were considered unlikely. Although other differentials could not be completely ruled out, DRE of unknown etiology was considered the most likely.

Based on these results, the epileptogenic zone of this case was not determined; therefore, the project team selected CCT as a suitable surgical procedure based on the following reasons: (1) epileptiform discharges distributed bilaterally; (2) unremarkable MRI; (3) severe seizure frequency that might result in decreased mental status; and (4) decreased QOL of the patient and the owner (Supplementary Data 1.1). As the final checkpoint to detect an identifiable epileptogenic focus, an intraoperative electrocorticography (ECoG) recording was planned immediately following the craniotomy. If the intraoperative ECoG recording clearly identified the epileptogenic focus, a different procedure such as a focal resection and/or multiple subpial transections would have been considered more suitable (Supplementary Data 1.1).

The owner was requested to record seizures including the types, frequency, and duration, as well as the visual analog scales (VAS; Supplementary Data 2) before and 3, 6, and 12 months after surgery. An attending neurologist (DH) evaluated the activities of daily living (ADL) (Supplementary Data 3) and performed follow-up EEG and MRI during the same period.

### Corpus Callosotomy

Corpus callosotomy (CCT) was performed at 12 months of age. General anesthesia was induced by propofol (6 mg/kg IV) and maintained with inhalation of isoflurane and oxygen. The cat was positioned in sternal recumbency in a Sphinx-like position. During surgery, electrocardiogram, oxygen saturation, end-tidal CO_2_ concentration, indirect blood pressure, rectal temperature, and urine production volume were monitored. Lactated Ringer's fluid was infused at 3–5 ml/kg/h, and a constant rate infusion (CRI) of remifentanil as intraoperative analgesia was performed at 20 μg/kg/h. After shaving and sterilization, an H-shaped scalp incision was made with the skin opened like a double door. Bilateral temporal muscles were incised along to the external sagittal crest and detached from the parietal bones, and then retracted bilaterally. A 3 cm (left–right) × 4 cm (rostral–caudal) rectangular-shaped craniotomy straddling the left and right of the parietal bone was performed. The first four burr holes were made on each corner of the rectangle with the rostral pair at the level of the caudal end of the frontal bone and the caudal pair approximately 1.5 cm rostral to the external occipital protuberance. The craniotomy was completed by connecting these burr holes to expose the underlying cerebral surface of the parietal lobes. In the process of connecting the burr holes, the craniotomy lines connecting the two rostral and the two caudal burr holes, i.e., transverse craniotomy lines, had widened areas across the midline in order to visualize the underlying dorsal sagittal sinus well. The removed bone fragment was wrapped with saline-filled gauze and saved for the closure.

After the craniotomy, epidural ECoG was recorded. A 1.5 × 1.5 cm grid-type silicon-sheet electrode with nine exploration electrodes was placed on the dura covering each hemisphere's parietal lobe. On ECoG, bilaterally synchronized spike-containing burst-suppression was recorded (Supplementary Data 1.4).

As ECoG did not aid to identify the epileptogenic zone, a sagittal sinus-based U-shaped dural incision was made on the left hemisphere and inverted to the right side. The left hemisphere was gently retracted laterally with neurosurgical putties, spatula, and shafts of suction to widen the longitudinal fissure. By peeling off the cingulate gyrus on both sides under the falx gently, the bright white CC was exposed (Supplementary Video 2).

The CC was bisected using bipolar cautery and suction from the caudal, the splenium, to the rostral, the genu. During the bisecting of the CC, the great cerebral vein, where the confluence of the vein of the corpus callosum and internal cerebral vein, was observed at the caudal end of the splenium. The third ventricle was also observed under the body. During the approach of the genu, a cortical vessel on the cingulate gyral surface was damaged, and the hemostasis manipulation and the subsequent brain swelling made it difficult to identify the genu. Therefore, the rostral dissection was abandoned (Supplementary Video 2). Due to the brain swelling, 1 g/kg of mannitol was intravenously administered over 15 minutes.

The dura was sutured with 6-0 polydioxanone after confirming normalized brain volume and no bleeding from the intracranial surgical field. Then, a post-callosotomy intraoperative ECoG was recorded; Although large spikes were still synchronized on both hemispheres, small independent spikes, not seen pre-CCT, were observed from each hemisphere (Supplementary Data 1.3). After recording ECoG, the stored bone fragment was returned and fixed with polymethylmethacrylate. Temporal muscles and scalp were sutured routinely. A percutaneous endoscopic gastrostomy (PEG) tube was placed to facilitate postoperative feeding.

The cat recovered from anesthesia uneventfully, and the patient's conscious level had moderately improved compared with the preoperative state. However, facial twitching was observed intermittently until the next day. As postoperative analgesia, CRI of fentanyl (2 μg/kg/h to tapered) was infused for 12 h after surgery, and a fentanyl patch (4.2 mg/head) was applied for 3 days. Although cefmetazole (25 mg/kg q2–12h IV) had been used from intra- to postoperative 12 h, the cat showed fever (~40°C) with mild neutrophilia (WBC 22,000 /μl) on the next day. Because bacterial meningitis was a potential concern, meropenem (25 mg/kg, CRI for 30 min q24h) for 3 days and enrofloxacin (5 mg/kg q24h PO) for 5 days were administered. The fever ceased within 4 h after initiation of meropenem, after that fever and leukocytosis had never been observed remaining the cause being unidentified.

The day after surgery, the cat was able to maintain a sitting position and eat wet food on his own, with the nutritional requirement maintained using a PEG tube. Furthermore, on the postoperative day (POD) 3, even though mild right-sided hemiparesis was observed, the cat became ambulatory and was able to voluntarily urinate and defecate. The PEG tube was removed at POD 10, and the cat was discharged from the hospital at POD 16. During the postoperative hospitalization, 5 myoclonic seizures were observed, while there were 12 seizure-free days. Neurological findings at the discharge time were slightly somnolence, mild disorientation, ambulate but generalized ataxia with postural reaction deficits in all limbs, and loss of biliteral menace response. Phenobarbital (3.5 mg/kg q12 h PO) and DZP (0.5 mg/kg q12 h PO) were continued throughout the hospitalization.

### Outcome

**Figure 3:**
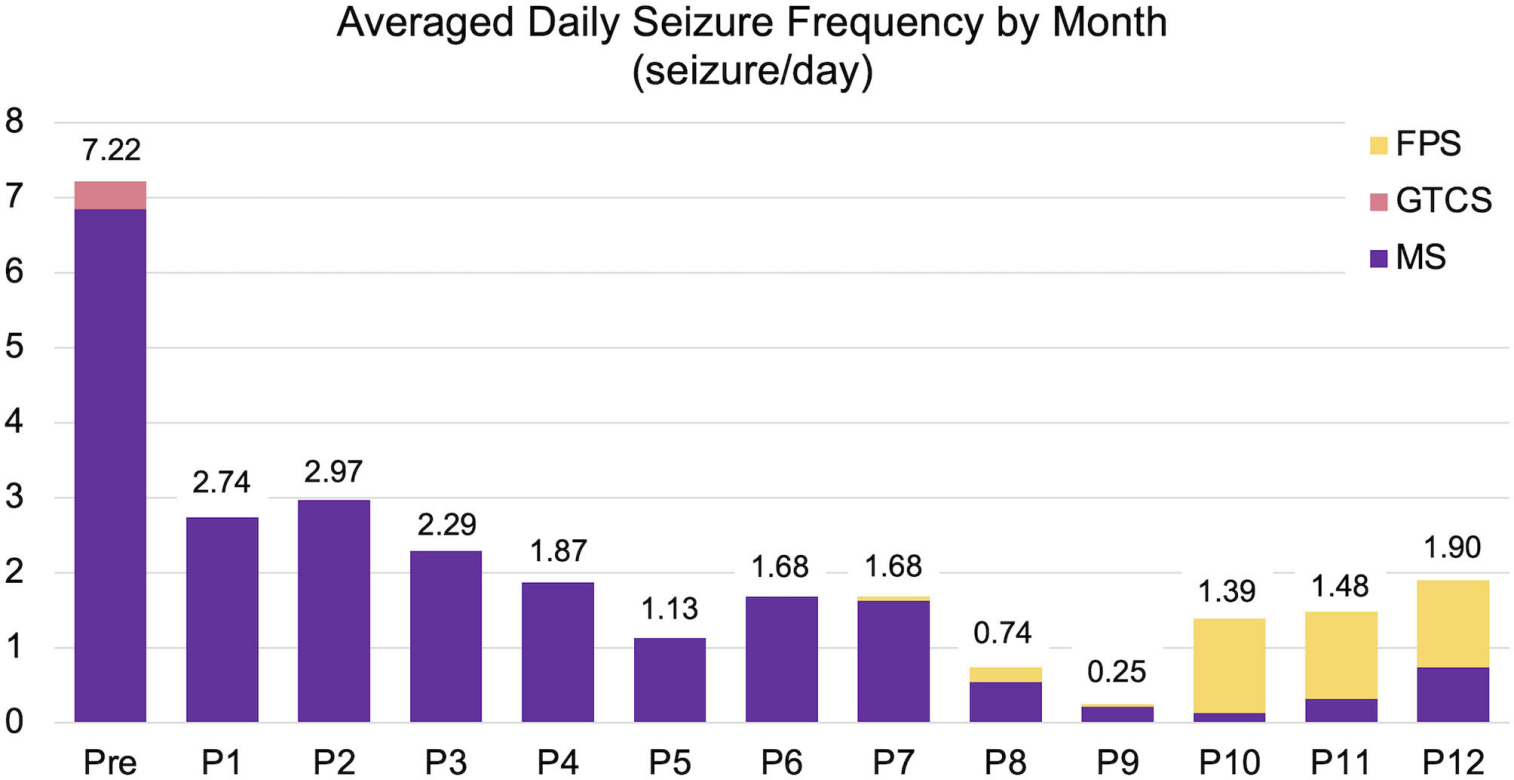
Changes in averaged daily seizure frequency (seizure/day) for preoperative (Pre) to postoperative 12 months (P1–P12). The preoperative seizure frequency (Pre; 7.22 sz/d) was shown as the average for 3-months before surgery. The average one-year postoperative seizure frequency was 1.7 sz/d (76% reduction). FPS (yellow), fencing posture seizures; GTCS (pink), generalized tonic-clonic seizures; MS (purple), myoclonic seizures including generalized, facial myoclonic, and short tonic seizures.

After discharge, the cat was routinely consulted at a local clinic every 2–4 weeks, and presented to the teaching hospital at 3, 6, and, 12 months after surgery for rechecks and follow-up EEG and MRI. Antiseizure drug therapy with PB and DZP were maintained for the 1-year follow-up period. The neurological status, mild cognitive impairment, ataxia with decreased postural reactions, loss of bilateral menace response, remained unchanged for the 1-year follow-up period. Mental status fluctuated from alert to somnolent depending on the time of the visit or seizure frequency. Changes of seizure types were observed postoperatively: GTCS had completely disappeared, and generalized myoclonic seizures changed to momentary facial myoclonic seizures and shorter tonic seizures. On the other hand, a new seizure type manifesting as the initial left arm-extended fencing posture (fencing posture seizure; FPS) followed by contralateral (right) myoclonic or clonic seizures (Supplementary Video 3) emerged 7 months postoperatively. Sequential changes in seizure frequency, VAS, and ADL from the preoperative time to the 1-year follow-up are shown in Figure 3, Table 1, and Supplementary Data 3, respectively. Seizure frequencies of all seizure types were markedly decreased (7.2–1.7 sz/d; 76% reduction); however, 1–2 sz/d in average either myoclonic seizures or FPS, or both remained unchanged through the 1-year follow-up period.

**Table 1:** Result of the visual analog scale (VAS).

	**Definition of 0/100**	**Pre**	**Post 3m**	**Post 6m**	**Post 12m**	Improved %^*^
Freq. Sz day	free/worst (everyday)	100	22	20	16	81
Freq. Myoclonic Sz^§^	free/worst	90	72	76	76	17
Duration of Myoclonic Sz^§^	0sec/2min^†^	100	13	11	17	86
Freq. GTCS	free/worst	69	0	0	0	100
Freq. Cluster Sz	free/worst	70	48	45	33	40
Freq. SE (≥5min)	free/worst	0	0	0	0	–
Severity of Sedation	none/comatose	78	37	48	48	43
Severity of Ataxia	none/non-ambulate	100	34	36	50	60
Degree of Activity	active/worst	84	18	37	27	67
Appetite^¶^	aphasia/polyphasia^¶^	25	80	32	79	128^¶^
Caregiver burden of Medication	not bothered/ extremely frustrated	84	80	100	87	−6
QOL of the animal	excellent/worst	49	16	18	24	60
QOL of the owner	excellent/worst	70	25	45	46	45
Satisfaction with surgery	satisfied/regret	NA	0	0	0	100

*The VAS sheet is shown in Supplementary Data 2. Values are actual measurements (mm) except the improved percentage. Pre, preoperative; Post 3, 6, and 12m; postoperative 3, 6, and 12 months; Freq., frequency; Sz, seizures; GTCS, generalized tonic-clonic seizures; SE, status epilepticus; QOL, quality of life; NA, not applicable*.

* *Improved percentage (%) = 100—{(average of 3 postoperative values) × 100 / preoperative value}*.

§ *Myoclonic seizures included shorter tonic seizures*.

† *Maximum value (100 mm) was defined as 2 min by the owner*.

¶ *Improved percentage of appetite was calculated as 50 mm indicated normal appetite (100%); thus, a 25 mm of preoperative value indicated 50% and a 64 mm of the average of postoperative values indicated 128%. Because the value of preoperative was not applicable, 0 mm of postoperative values were considered as 100% satisfied*.

Follow-up scalp EEGs showed clearly decreased spike frequency. Although a few spikes were observed in a hemisphere only, most were still synchronized bilaterally (Figures 1B–D). Follow-up MRI at 3, 6, and 12 months after surgery revealed the bisected CC with the remaining genu, loss of caudal part of the left cingulate gyrus, and old hemorrhagic and a necrotic lesion in the left cingulate gyrus likely related to intraoperative tissue handling as well as vascular damage (Figure 2B).

## Discussion

This is the first report of CCT in a clinical feline case with DRE of unknown cause. Although very short myoclonic seizures and occasionally FPS remained, a meaningful reduction in seizure frequency and duration was achieved for the 1-year follow-up period. Furthermore, it is also important that the mental status, activity, and the QOL of both patient and owner improved substantially after surgery.

Corpus callosotomy is categorized as a palliative surgery together with neuromodulations such as vagus nerve stimulation (VNS) and deep brain stimulation (DBS) in human epilepsy. Palliative epilepsy surgeries are generally applied to various types of generalized seizures and/or focal seizures evolving to GTCS with unknown, multiple, or bilateral epileptogenic zone(s). There are many comparative studies between CCT and VNS for human generalized DRE. In general, CCT is more invasive and requires an intracranial procedure with a higher risk of complications; however, CCT is superior in reducing seizures and fast acting compared with VNS. One study that compared CCT and VNS for refractory generalized seizures showed the percentages of patients who had a ≥50% seizure reduction was 79 and 40%, respectively (9). In the same study, however, the complication rate of CCT (21%) was higher than that of VNS (8%). In one systematic review focusing on atonic seizures and drop attacks only, ≥50% seizure reduction was 85.6% and 57.6% for CCT and VNS, respectively (10).

There is no information regarding the clinical outcome of CCT in veterinary patients. Corpus callosotomy in the present case achieved a 100% reduction in GTCS and a ≥75% reduction for all seizure types. The preoperative neurological status of this case was difficult to assess accurately because the severely disabled condition, i.e., abasia-astasia and stuporous mental status, which were considered to be attributed to multiple factors including the underlying condition of continuous seizure activities, the consequence of seizure activities, and the adverse effect of multiple ASDs. There was no major complication except a transient right-dominant tetraparesis after surgery. Postoperative improvement of neurological and QOL status was more conspicuous, which brought high satisfaction to the owner. The owner showed the greatest pleasure and excitement when the cat stood up, walked, ate, and excreted himself after surgery, as shown in VAS and ADL improvement. In fact, CCT efficacy in humans is observed not only in seizure reduction but also in overall daily function, behavior, cognition, and intelligence in over half of patients (2). The authors considered that the brain dysfunction associated with ongoing seizure activities restored its function relatively well along with decreased seizure episodes following CCT.

The CCT surgical procedure in cats has not been reported in the veterinary literature. Therefore, we performed it based on reports in dogs (4) and humans (2). Although the incomplete dissection may be the cause of the residual seizures, most of the CC, including splenium, was clearly visualized and easily bisected compared with the authors' limited experience of CCT in dogs (unpublished information). On the other hand, a caudorostoral approach to the genu was challenging in this case due to the intraoperative injury to the cingulate gyrus and subsequent cerebral edema. More rostrally extended craniotomy allowing better visualization of the genu and rostral cerebral artery might be necessary to bisect the genu as in human anterior CCT. A stereotaxic bisection of the CC was used (7) in the previous experimental CCT studies in feline seizure models (5, 6, 8). This stereotaxic method with wire seemed easier to perform; however, the CC was not visually identified. With this stereotaxy-guided blind dissection, the surrounding structures, including cingulate gyrus and vessels were not protected and they might have been damaged. The authors elected not to utilize this technique given this potential lethal risk. Other feline experimental studies of binocularity had described CCT procedure with the aid of stereotaxic frame and surgical microscope (11, 12). Those studies, however, did not aim to inhibit seizures, and had bisected caudal CC mainly. In human CCT, neuro-endoscopic CCT and radiosurgical or laser interstitial thermal therapeutic CCT have been recently developed to decrease the invasiveness and the complication rates (2, 3, 9). If those types of equipment would be available in veterinary medicine, the surgical technique of CCT may change in the future.

Interestingly, in the present case, the seizure type showing the left arm-extended fencing posture at the onset, which is considered a specific seizure sign of the contralateral frontal lobe in humans, appeared after CCT. This seizure type suggested that (at least one of) the epileptogenic zone(s) of the present case localized in the right frontal lobe. Perhaps, the remaining genu may be the cause of the seizure activity transition from the right frontal lobe to the left hemisphere. FPS may also be an altered manifestation of preoperative GTCS. The recognition of laterality of epileptogenicity after CCT in human patients with secondarily generalized seizures without distinct laterality is considered an advantage of CCT, which will be an indicator for subsequential resection surgery (1, 2, 13). Although we do not plan the second resection surgery for the current case at present, it may be performed if seizures increase and/or the owner requests it in the future.

As mentioned above, CCT is often compared with VNS in humans for various factors. For veterinary patients in particular, the size, weight, and costs of the implantable pulse generator (IPG) of VNS (and also DBS) would be a considerable limitation for patients and owners, and there is no specific IPG for small veterinary patients. Currently available IPGs for human patients are quite large and heavy for cats or small breed dogs, and likely cost-prohibitive (10,000–15,000 USD). Furthermore, potential complications such as infection or malfunction of the device during long-term implantation must be taken into consideration. Therefore, we expect that CCT may be a reasonable epilepsy surgery for veterinary patients, although pros and cons of CCT and VNS will become clear in the future.

In conclusion, this is the first report of CCT in a client-owned feline patient with DRE describing relatively positive outcome. Although it will take a long time and the accumulation of both positive and negative cases to establish the utility of CCT in veterinary patients with DRE, the authors hereby present epilepsy surgery as one of the new options for the treatment of epilepsy in veterinary medicine and declare the dawn of a new era of epilepsy treatment in veterinary medicine.

## Data Availability Statement

The original contributions presented in the study are included in the article/Supplementary Material, further inquiries can be directed to the corresponding author.

## Ethics Statement

The animal study was reviewed and approved by the Ethics Committee of the Veterinary Medical Teaching Hospital, Nippon Veterinary and Life Science University (accession no. H30-1). Written informed consent was obtained from the owners for the participation of their animals in this study.

## Author Contributions

DH and SK: conception, design, and writing the draft. All authors data acquisition and analysis, revising the draft, and approval of the final manuscript.

## Funding

This clinical trial was supported by a Grant-in-Aid for Scientific Research (A) of JSPS KAKENHI Grant Number 17H01507.

## Conflict of Interest

The authors declare that the research was conducted in the absence of any commercial or financial relationships that could be construed as a potential conflict of interest.

## Publisher's Note

All claims expressed in this article are solely those of the authors and do not necessarily represent those of their affiliated organizations, or those of the publisher, the editors and the reviewers. Any product that may be evaluated in this article, or claim that may be made by its manufacturer, is not guaranteed or endorsed by the publisher.

## Supplementary Material

Supplementary Data 1 Supplementary explanations and results for (1) the Project team and clinical trial, (2) scalp EEG, (3) MRI, and (4) intraoperative ECoG.

Supplementary Data 2 Visual analog scale sheet.

Supplementary Data 3 Activities of daily living sheet and the results.

Supplementary Video 1 Videos of the preoperative seizures.

Supplementary Video 2 Video of the surgical procedure of corpus callosotomy.

Supplementary Video 3 Videos of the postoperative seizures.

## References

[1] Graham D Tisdall MM Gill D. Corpus callosotomy outcomes in pediatric patients: A systematic review. Epilepsia. (2016) 57:1053–68. 10.1111/epi.13408 27237542

[2] Asadi-Pooya AA Sharan A Nei M Sperling MR. Corpus callosotomy. Epilepsy Behav. (2008) 13:271–8. 10.1016/j.yebeh.2008.04.020 18539083

[3] Vaddiparti A Huang R Blihar D Du Plessis M Montalbano MJ Tubbs RS. The evolution of corpus callosotomy for epilepsy management. World Neurosurg. (2021) 145:455–61. 10.1016/j.wneu.2020.08.178 32889189

[4] Bagley RS Baszler TV Harrington ML Pluhar GE Moore MP Keegan RD. Clinical effects of longtudinal division of the corpus callosum in normal dogs. Vet Surg. (1995) 24:122–7. 10.1111/j.1532-950X.1995.tb01306.x 7778251

[5] Wada JA Nakashima T Kaneko Y. Forebrain bisection and feline amygdaloid kindling. Epilepsia. (1982) 23:521–30. 10.1111/j.1528-1157.1982.tb05438.x 7140664

[6] Fukuda H Wada JA Riche D Naquet R. Role of the corpus callosum and hippocampal commissure on transfer phenomenon in amygdala-kindled cats. Exp Neurol. (1987) 98:189–97. 10.1016/0014-4886(87)90083-5 3653330

[7] Magni F Melzack R Smith CJ. A stereotaxic method for sectioning the corpus callosum in cat. Electroencephalogr Clin Neurophysiol. (1960) 12:517–8. 10.1016/0013-4694(60)90035-3 14419872

[8] Wada JA Sato M. The generalized convulsive seizure state induced by daily electrical stimulation of the amygdala in split brain cats. Epilepsia. (1975) 16:417–30. 10.1111/j.1528-1157.1975.tb06069.x 1183418

[9] Nei M O'Connor M Liporace J Sperling MR. Refractory generalized seizures: Response to corpus callosotomy and vagal nerve stimulation. Epilepsia. (2006) 47:115–22. 10.1111/j.1528-1167.2006.00377.x 16417539

[10] Rolston JD Englot DJ Wang DD Garcia PA Chang EF. Corpus callosotomy versus vagus nerve stimulation for atonic seizures and drop attacks: A systematic review. Epilepsy Behav. (2015) 51:13–7. 10.1016/j.yebeh.2015.06.001 26247311 PMC5261864

[11] Yinon U Chen M Gelerstein S. Binocularity and excitability loss in visual cortex cells of corpus callosum transected kittens and cats. Brain Res Bull. (1992) 29:541–52. 10.1016/0361-9230(92)90121-D 1422852

[12] Payne BR Elberger AJ Berman N Murphy EH. Binocularity in the cat visual cortex is reduced by sectioning the corpus callosum. Science. (1980) 207:1097–9. 10.1126/science.7355278 7355278

[13] Hwang ST Stevens SJ Fu AX Proteasa S V. Intractable generalized epilepsy: therapeutic approaches. Curr Neurol Neurosci Rep. (2019) 19:16. 10.1007/s11910-019-0933-z 30806817

