# Supplementary material for: Case Report: Corpus Callosotomy in a Cat With Drug-Resistant Epilepsy of Unknown Cause
Source: Front Vet Sci. 2021 Sep 29;8:745063. doi: 10.3389/fvets.2021.745063 (PMC8511771; doi:10.3389/fvets.2021.745063)
Supplement: Supplementary Data 1 — Supplementary explanations and results for (1) the Project team and clinical trial, (2) scalp EEG, (3) MRI, and (4) intraoperative ECoG. [file Data_Sheet_1.docx]

Supplementary Data 1

# The project team of veterinary epilepsy surgery and the clinical trial, inclusion criteria, and selection of surgical procedure

The research project entitled “Development of epilepsy surgery in small animal veterinary medicine” was planned by the first author (DH) as the principal investigator and was financially supported by the Grant-in-Aid for Scientific Research (A) of Japan Society for the Promotion of Science (JSPS) KAKENHI (Grant number: 17H01507) between 2017–2021. The project team consisted of 4 board-certificated veterinary neurologists and a neuropathologist as principal researchers, some colleagues of each principal researcher as collaborators, and some medical doctors (epileptologists and neurosurgeons referred in the Acknowledgement section in the main text) as advisers.

As a part of this project, the clinical trial of epilepsy surgery (prospective case study) is ongoing, and the case in this report and a previously published canine case that underwent vagus nerve stimulation (Hirashima et al. 2021) are early cases of the trial. In this clinical trial, the candidate with drug-resistant epilepsy (DRE) for epilepsy surgery was defined as: 1) canine or feline patient diagnosed with idiopathic, unknown cause or structural epilepsy with ≥ Tier II level of IVETF criteria (i.e. already ruled out reactive seizures and performed pre-post prandial total bile acid measurement, MRI, and CSF analysis); 2) canine or feline patients with epilepsy who could not be controlled seizures < 1 seizure every 3 months despite using 3 out of 4 available antiseizure drugs (ASD) adequately (phenobarbital, zonisamide, bromide, and levetiracetam for dogs; phenobarbital, levetiracetam, zonisamide, and diazepam for cats); 3) canine or feline patient who has markedly decreased QOL due to unacceptable adverse effects of ASDs or frequent seizure activity itself. In this inclusion criteria, both idiopathic (unknown) and structural epilepsies are acceptable, but structural epilepsy is limited to the lesion which is non-progressive pathology; for example, a patient with structural epilepsy caused by past traumatic lesion will be accepted, while a patient with brain tumor is rejected. Additionally, the blood levels of phenobarbital, bromide, and zonisamide have to be measured and achieved (once at least) certain (close to maximum) concentration: 30–35 µg/ml for phenobarbital, > 1.0 mg/dl for bromide, and 30–40 µg/ml for zonisamide. Upon an application of a candidate case by a veterinarian, 4 veterinary neurologists of the project team evaluate whether the candidate is true DRE patient or not (if not, the project team return the patient with the reason and recommendation for the further treatment options), and discuss additionally required presurgical evaluations such as video monitoring for complete seizure semiology, scalp and/or intracranial electroencephalography (EEG) with/without video monitoring, advanced structural and functional MRI by ≥ 1.5T magnet. If the patient is accepted for epilepsy surgery, a detailed explanation of the clinical study is given to the owner and obtained informed consent. Medical fees, including the fee of required presurgical investigations, surgery, hospitalization, and follow-up investigations (EEG and MRI at 3, 6, and 12 months after the surgery), are waived after the owner consents to participate in the clinical trial.

When all required presurgical data of the patient (minimum ictal video, EEG, and high-field MRI findings) have been collected, the project team discuss and chose a suitable surgical procedure for the patient referred to the human’s strategy (Benbadis et al. 2018). Our provisional algorithm for selecting surgical procedures is shown in **Supplementary Figure 1**. In the present case, preoperative seizure semiology and scalp EEG findings showed generalized myoclonic and tonic-clonic seizures in addition to being an urgent case; therefore, corpus callosotomy was chosen (shown in the red line on **Supplementary Figure 1**).

**Supplementary Figure 1.** Provisional algorithm for selecting surgical procedures in this clinical study. The course of the case cat in the main text is shown in red arrows (GS–Urgency–CCT). Blue and green arrows also indicate the courses of previously reported cases (Blue; Hirashima et al. 2021 and Green; Hasegawa et al. 2021– preclinical study). CCT, corpus callosotomy; EEG, electroencephalography; FS, focal seizures; GS, generalized seizures; iEEG, intracranial EEG; MRI +/–, presence or absence of the epileptogenic lesion(s) on magnetic resonance imaging; MTL, mesial temporal lobe; MST, multiple subpial transection; Sz, seizure; TL-AH, temporal lobectomy and amygdalohippocampectomy; ViEEG, long-term video-iEEG monitoring; VNS, vagus nerve stimulation. *Others means the reason(s) to avoid ViEEG monitoring due to a patient having another pathology other than epilepsy, a patient cannot be stable at hospitalization, or the owner who does not accept, etc.

# Scalp EEG

Preoperative and follow-up scalp EEGs were performed under sedation with dexmedetomidine (20 µg/kg, IV). After the cat became sedative, subdermal stainless wires were penetrated using a 25-G needle on each electrode part of the scalp and were pinched by clip-tip electrodes. Electrode positions were F3 (left frontal), F4 (right frontal), C3 (left central), C4 (right central), T3 (left temporal), T4 (right temporal), O1 (left occipital), O2 (right occipital), Fz (mid frontal), Cz (mid central), and Pz (mid occipital) according to the previously suggested arrangement (Hasegawa Vet J 2016). EEG was recorded with a digital EEG system (Neurofax EEG-1200; Nihon Kohden, Tokyo, Japan). Recording conditions were as follows; sampling rate = 1,000 Hz, high-cut filter = 60 Hz, time constant = 0.1–0.3, and AC filter = on. Body temperature was maintained at 37–38 ºC using hot-water bags. Although montage could be changed suitably in the recorded EEG (i.e. re-montage), reviewing montages were referential (monopolar; reference was average referential or digital zero) and longitudinal bipolar derivations as shown in Figure 1 in the main text. Electrocardiograms were also recorded with the EEG system. Recording times ranged from 20–40 minutes among each recording.

# MRI

Preoperative and follow-up MRIs were performed with 3.0T superconducting MRI unit (Signa HDxt; GE Healthcare, Tokyo, Japan) under general anesthesia, which was induced by intravenous propofol and maintained with inhalation of isoflurane and oxygen. An 8-channel human knee coil was used as RF coil. Scanned sequences were as follows; 3D-T2 cube (FSE, TR/TE = 3,200/78.5 ms, FOV = 15 × 15 cm, slice thickness = 0.6 mm, matrix = 256 × 256, NEX = 1), 3D-T1 SPGR (SPGR, TR/TE/TI = 6.5/3.1/450 ms, FOV = 15 × 15 cm, matrix = 256 × 192, NEX = 1) with and without gadodiamide (0.1 mmol/kg, IV; Omniscan; GE Healthcare, Tokyo, Japan), transverse T2-weighted (FSE, TR/TE = 7,000/85 ms, FOV = 15 × 15 cm, slice thickness = 2.0 mm, slice gap = 0.5 mm, matrix = 384 × 288, NEX = 1), T2-fluid-attenuated inversion recovery (FSE, TR/TE/TI = 11,000/140/2,400 ms, FOV = 15 × 15 cm, slice thickness = 2.0 mm, slice gap = 0.5 mm, matrix = 256 × 192, NEX = 2), T2*-weighted (GRE, TR/TE = 740/18 ms, FOV = 15 × 15 cm, slice thickness = 3.0 mm, slice gap = 0.5 mm, matrix = 320 × 192, NEX = 2), diffusion-weighted imaging (FSE propeller, TR/TE = 8,000/72 ms, b = 1,000, 3 axes, FOV = 15 × 15 cm, slice thickness = 2.0 mm, slice gap = 0.5 mm, matrix = 128 × 128, NEX = 1), and diffusion tensor imaging (EPI, TR/TE = 8,000/94 ms, b = 1,000, 15 axes, FOV = 15 × 15 cm, slice thickness = 2.4 mm, slice gap = 0.5 mm, matrix = 128 × 128, NEX = 2). At the preoperative scanning, apparent diffusion coefficient and fractional anisotropy values were calculated and mapped by the internal software of the MRI system; however, there were no specific findings. And also, voxel-based morphometry for gray and white matters was preoperatively performed using 3D-T1 data, but no statistically significant area was detected. Although dynamic susceptibility contrast perfusion-weighted imaging and single point resolved spectroscopy MR spectroscopy were tried at the preoperative scan, the results of those were not available. Fiber tracking by diffusion tensor imaging data was used for confirmation of the bisected corpus callosum at the follow-up MRIs (**Supplementary Figure 2**).


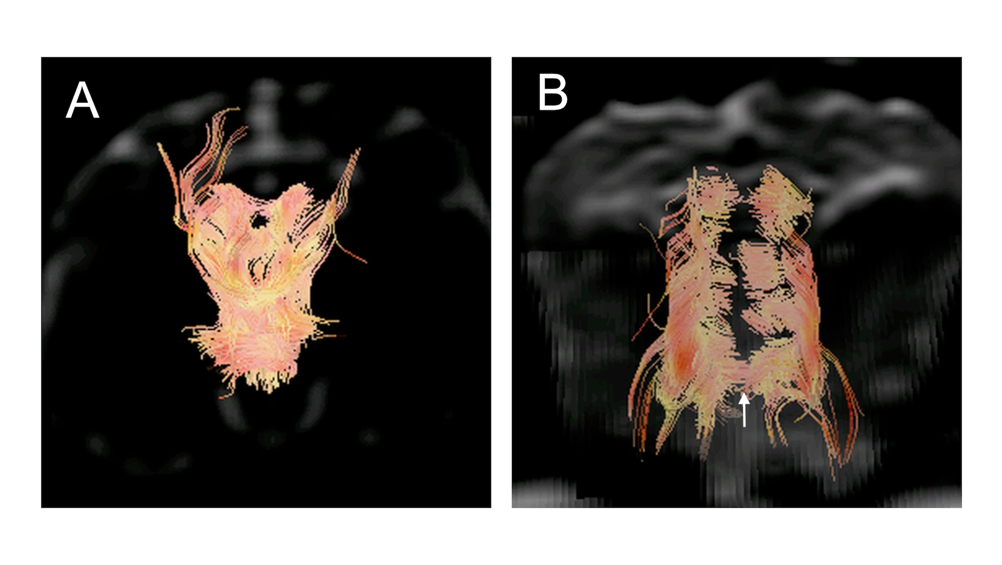


**Supplementary Figure 2.** Oblique dorsal view of preoperative (A) and postoperative (B) fiber tracking of the corpus callosum of the case cat. In the postoperative image (B), disconnection of the callosal fibers is confirmed except the rostral part (the genu; arrow).

# Intraoperative ECoG

Electrocorticography (ECoG) was recorded intraoperatively before and after corpus callosotomy using the same equipment for scalp EEG (Neurofax EEG-1200; Nihon Kohden, Tokyo, Japan). During the ECoG recording, the concentration of isoflurane was decreased to 0.5% to obtain reasonable cortical activities, but CRI of remifentanil was continued without changing the dose (20µg/kg/h). A 1.5 cm x 1.5 cm grid having 9 electrodes (ø 3.0 mm Pt-hat electrode; 5mm interval for each) (TY217-033 ECoG electrode, Unique Medical, Tokyo, Japan) was placed on the dura mater covering the parietal lobe in each hemisphere. Electrode positions and their number are illustrated in Supplementary Figure 3. Monopolar derivation was used for real-time monitoring; the referential screw electrode was placed on the external occipital protuberance. Recording conditions were the same as the abovementioned scalp EEG; however, the sensitivity (gain) had to be reduced to 75 µV for monitoring or reviewing ECoG due to its higher amplitude. Recording time was around 5 minutes for each. Recorded intraoperative ECoGs before and after callosotomy are shown in **Supplementary Figure 3**.


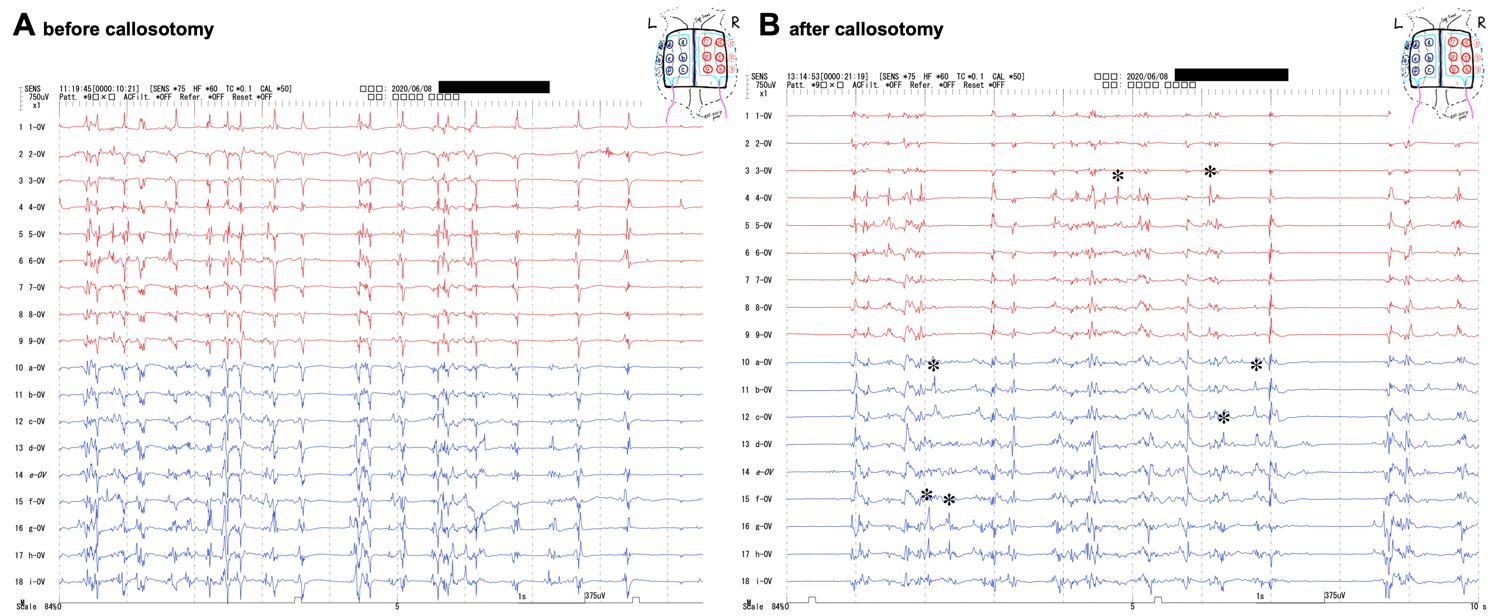


**Supplementary Figure 3.** Intraoperative electrocorticograms (ECoG) of before (A) and after (B) corpus callosotomy. For agreement with the surgical field of view from the operator, the electrodes of the right hemisphere were displayed at the upper traces (Ch 1–9; reds), while those of left were at the lower traces (Ch a–i; blues). Before callosotomy (A), all spikes were synchronized bilaterally. After callosotomy (B), some small but desynchronized spikes (*) had been observed and the amplitude of the right hemisphere were decreased comparing with the left.

# References

Hirashima J, Saito M, Igarashi H, Takagi S, Hasegawa D. Case report: One-year follow-up of vagus nerve stimulation in a dog with drug-resistant epilepsy. *Front Vet Sci.* (2021) **8**:708407. doi: 10.3389/fvets.2021.708407

Benbadis SR, Geller E, Ryvlin P, Schachter S, Wheless J, Doyle W, Vale FL. Putting it all together: Options for intractable epilepsy–An update algorithm on the use of epilepsy surgery and neurostimulation. *Epilepsy Behav.* (2018) **88**:33-38. doi: ﻿10.1016/j.yebeh.2018.05.030

Hasegawa D, Asada R, Hamamoto Y, Yu Y, Kuwabara T, Mizoguchi S, Chambers JK, Uchida K. Focal resection and hippocampectomy in a cat with drug-resistant structural epilepsy. Front Vet Sci. (2021) **8**:719455. doi: 10.3389/fvets.2021.719455

Hasegawa D. Diagnostic techniques to detect the epileptogenic zone: Pathophysiological and presurgical analysis of epilepsy in dogs and cats. *Vet J.* (2016) **215**:64-75. doi: 10.1016/j.tvjl.2016.03.005
