# Supplementary material for: Case Report: Corpus Callosotomy in a Cat With Drug-Resistant Epilepsy of Unknown Cause
Source: Front Vet Sci. 2021 Sep 29;8:745063. doi: 10.3389/fvets.2021.745063 (PMC8511771; doi:10.3389/fvets.2021.745063)
Supplement: Supplementary Data 2 — Visual analog scale sheet. [file Data_Sheet_2.docx]

Supplementary Data 2

# Visual analog scale (VAS)

In order to evaluate the owner’s subjective assessment for various aspects regarding the effect of epilepsy surgery to the patient and owner him/her self, the owner was requested to record the visual analog scale (VAS) shown below preoperative and postoperative at 3, 6, and 12 months after surgery. This standardized VAS sheet was used for all patients included in the clinical trial of epilepsy surgery with some arrangements for each patient if necessary. For example, because the feline case presented here showed no focal seizures initially but showed two types of generalized seizures, the terms of ‘focal seizures’ and ‘generalized seizures’ in the standardized VAS sheet were rephrased into ‘myoclonic seizures’ and ‘tonic-clonic seizures’, respectively. The owner was requested to mark on the scale of 100 mm horizontal line for each evaluation item. The researcher collected the VAS sheet at each follow-up consultation and measured the distance of mark of each evaluation item. The results of VAS are shown in Table 1 in the main manuscript.

**Visual Analog Scale (ver. 3)**

Date：mm/dd/yy 　　　（preoperative・postoperative　　　　months）

Owner’s name：　　　　　　　　　　　 Dog’s or Cat’s name： d

Please mark a vertical line on the horizontal line at the point which most accurately describes your feelings or impression to each question.

1. Frequency of seizure day

None　　　　　　　　　　　　　　　　　　　　 High

　　　　　　mm

1. Frequency of Myoclonic Seizures (originally ‘focal seizures’)

None　　　　　　　　　　　　　　　　　　　 　High

　　　　　　mm

1. Duration of Myoclonic Seizures (originally ‘focal seizures’)

None　　　　　　　　　　　　　　　　　　　　 High

　　　　　　　　　　mm

1. Frequency of Generalized Tonic-Clonic Seizures

None　　　　　　　　　　　　　　　　　　　　 High

　　　　　　mm

1. Frequency of Cluster Seizures

None　　　　　 　　　　　　　　　　　　　　　High

　　　　　　　　　　　　　　　　　　　　　　　　　　　　　　　　　　mm

1. Frequency of Status Epilepticus (≥ 5 minutes)

None High

mm

1. Severity of Sedation (consciousness)

Normal　　　　　　　　　　　　　　　　　　 Always Sleeping

　　　　　　　　　　　　　　　　　　　　　　　　　　　　　　　　　　mm

1. Severity of Ataxia

None　　　　　　　　　　　　　　　　　　 Hard to walk and stand

　　　　　　　　　　　　　　　　　　　　　　　　　　　　　　　　　　mm

1. Degree of Activity/Daily life

Active (same as before the onset of epilepsy)　　 Worst

　　　　　　　　　　　　　　　　　　　　　　　　　　　　　　　　　　mm

1. Appetite

None　　　　　　　　　　　　　　　　　　　 Too much

　　　　　　　　　　　　　　　　　　　　　　　　　　　　　　　　　　mm

1. Burden of medication on your dog/cat

No drugs　　　　　　　　　　　　　　　　　 　　 Burdensome

　　　　　　　　　　　　　　　　　　　　　　　　　　　　 　　　　　　mm

1. The quality of life in your dog/cat

Excellent　　　　　　　　　　　　　　 　　　 Worst (considering euthanasia)

　　　　　　　　　　　　　　　　　　　　　　　　　　　　　　　　　　mm

1. The quality of life in the owner (yourself)

Excellent　　　　　　　　　　　　　　 　　　 Worst

　　　　　　　　　　　　　　　　　　　　　　　　　　　　　　　　　　mm

1. Satisfaction with the surgery

Satisfied Regret

　　　　　　mm
