# Supplementary material for: Case Report: Corpus Callosotomy in a Cat With Drug-Resistant Epilepsy of Unknown Cause
Source: Front Vet Sci. 2021 Sep 29;8:745063. doi: 10.3389/fvets.2021.745063 (PMC8511771; doi:10.3389/fvets.2021.745063)
Supplement: Supplementary Data 3 — Activities of daily living sheet and the results. [file Data_Sheet_3.docx]

Supplementary data 3

# Activities of daily living (ADL)

The researcher (attending doctor) scored the sheet of activities of daily living (ADL) shown below preoperative and postoperative at 3, 6, and 12 months. The results of those are shown under the score sheet as Supplementary Table 1. Postural changes, grooming, urination, posture, and gait improved ≥ 50 %, and the total score also increased by 57 points postoperatively.

**Score sheet of ADL for dogs and cats**

Date: dd/mm/yy 　　　　　 ID: 　　　　 Scorer:

| Activity | Score | Guidepost | Patient’s Score |
| --- | --- | --- | --- |
| 1. Eating | 15 | Can eat and drink voluntarily |  |
|  | 10 | Need partial assistance |  |
|  | 0 | Need total assistance (forced feeding) |  |
| 2. Changing positions | 10 | Can change voluntarily |  |
|  | 5 | Need partial assistance |  |
|  | 0 | Need total assistance |  |
| 3. Grooming | 10 | Can groom its whole body without difficulty |  |
|  | 5 | Can groom itself but the limited areas |  |
|  | 0 | Impossible |  |
| 4. Excretion activity | 10 | Can hold excretion posture itself |  |
|  | 5 | Need assistance to hold the posture |  |
|  | 0 | Excrete at bedridden |  |
| 5. Urination | 15 | Voluntary, without incontinence |  |
|  | 10 | Both voluntary and involuntary |  |
|  | 0 | Involuntary (need assist or expression) |  |
| 6. Defecation | 15 | Voluntary, without incontinence |  |
|  | 10 | Both voluntary and involuntary |  |
|  | 0 | Involuntary (need assistance or disimpaction) |  |
| 7. Posture | 10 | Can get up and keep the posture itself |  |
|  | 5 | Need an auxiliary tool to hold the prone position |  |
|  | 0 | Other than above |  |
| 8. Gait | 15 | Ambulate, voluntary |  |
|  | 10 | Need assist (e.g. sling) to walk  Can move voluntarily by creep |  |
|  | 0 | Non-ambulate, need total assistance |  |
| Total Score | | |  |

**Supplementary Table 1. Result of the activities of daily living (ADL)**

| Ability | Score range | Preope | Post 3m | Post 6m | Post 12m | Increased %* |
| --- | --- | --- | --- | --- | --- | --- |
| 1. Eating | 0, 10, 15 | 10 | 10 | 10 | 10 | 0 |
| 2. Postural changing | 0, 5, 10 | 0 | 10 | 10 | 10 | 100 |
| 3. Grooming | 0, 5, 10 | 0 | 5 | 5 | 5 | 50 |
| 4. Excretion activities | 0, 5, 10 | 5 | 10 | 5 | 5 | 17 |
| 5. Urination | 0, 10, 15 | 0 | 15 | 10 | 10 | 78 |
| 6. Defecation | 0, 10, 15 | 10 | 15 | 15 | 10 | 38 |
| 7. Posture | 0, 5, 10 | 0 | 10 | 10 | 10 | 100 |
| 8. Gait | 0, 10, 15 | 0 | 15 | 15 | 15 | 100 |
| Total score | 0 – 100 | 25 | 90 | 80 | 75 | 57 |

Values are actual score (point) except the increased percentage. * Increased percentage is calculated as followed: the score range of each item is transformed to 0–100%. The preoperative and average of 3 postoperative scores are also transformed, then the postoperative average % minus preoperative % is shown as the increased percentage on the table.
